# Barriers and facilitating factors to healthcare accessibility among Nepalese migrants during COVID-19 crisis in Japan: an exploratory sequential mixed methods study

**DOI:** 10.1186/s12889-023-16107-7

**Published:** 2023-06-24

**Authors:** Sushila Paudel, Aliza K C Bhandari, Stuart Gilmour, Hyeon Ju Lee, Sakiko Kanbara

**Affiliations:** 1 Graduate School of Nursing, University of Kochi, Kochi, Japan; 2 Graduate School of Public Health, St. Luke’s International University, Tokyo, Japan; 3 Department of Health Policy, National Center for Child Health and Development, Okura 2-10-1, Setagaya City Tokyo, 157-8535 Japan; 4 Kobe City College of Nursing, Kobe, Japan

**Keywords:** Barriers, Facilitators, COVID-19, Healthcare access, Migrants, Mixed methods, Nepalese, Japan

## Abstract

**Background:**

The COVID-19 pandemic has highlighted the need for global unity and timely access to healthcare for all including multilingual and intercultural societies. This study aimed to identify barriers to healthcare access due to the COVID-19 crisis among Nepalese migrants in Japan and explore ways to counter these barriers, both in routine and crisis situations.

**Methods:**

This study used an exploratory sequential mixed-methods study design. The researchers conducted 11 focus group discussions including 89 participants and an online survey involving 937 respondents. The integration of focus group discussions and logistic regression analysis from the survey was reported via a ‘joint display’.

**Results:**

Twenty-six themes on barriers to and six on facilitators of healthcare accessibility were identified by the focus group discussions among which 17 barriers like lack of knowledge of health insurance, language barriers, lack of hotline services, unawareness of available services, fear of discrimination etc. had significant association in our logistic regression analysis after adjusting for all confounders. Similarly, the only facilitator that had a significant impact, according to the multivariable logistic regression analysis, was receiving health information from Nepali healthcare professionals (OR = 1.36, 95% CI = (1.01 – 1.82), *p*-value < 0.05).

**Conclusion:**

The study suggests the need for a crisis information hub which could be coordinated by the Nepal embassy or concerned authorities, flexible policies for active deployment of Nepalese health workers and volunteers, accessible hotlines in the Nepali language, and incorporation of Nepali telehealth services in Japan.

**Supplementary Information:**

The online version contains supplementary material available at 10.1186/s12889-023-16107-7.

## Introduction

The COVID-19 pandemic has highlighted the need for global unity and timely access to healthcare for all including multilingual and intercultural societies [1–4]. Migrant workers are often particularly vulnerable, [5–7] and this vulnerability is heightened during disasters [8–11]. The COVID-19 pandemic has exacerbated the vulnerabilities of the migrants, [12] and in many countries they have been wrongly accused of spreading the virus and are subjected to stigmatization [8, 13–15]. Barriers to healthcare accessibility among migrants during the COVID-19 pandemic include language barriers; [16–18] unwillingness to ask for assistance due to fear of arrest and/or stigmatization, [19–22] privacy concerns, and cultural barriers [23]. These barriers were significant even before the pandemic, [24, 25] but have been detrimental during the crisis.

In Japan, the healthcare system is built upon a universal health insurance program that mandates enrollment of all including foreign residents, in either Employees' Health Insurance system (EHI), National Health Insurance system (NHI) or health insurance system for people above 75 years of age [26]. Those under 70 years old are responsible for paying 30% of the medical expenses except for children below three years, while the government covers the remaining 70%. The healthcare system also does not clearly differentiate between primary and secondary care and does not have a gate-keeper system that allows individuals the freedom to select their preferred medical facilities, which has resulted in shortages of general practitioners [27]. Foreign residents often face barriers to accessing healthcare as the country's healthcare system are generally structured without considering the needs of non-Japanese patients [28, 29]. These barriers can include language hurdles, cultural differences, lack of information on health and welfare services, and difficulty in applying for benefits [30, 31]. This can be particularly problematic during emergencies or disasters, when timely access to healthcare becomes even more critical. Additionally, the number of migrants in Japan has been increasing rapidly with Chinese, Korean, Vietnamese, Filipino, Brazilian, and Nepalese people making up the largest number of migrants [32]. Nepalese are the largest South Asian community in Japan and have seen a significant increase in their rate of migration over the past decade [33].

There are currently a total of 97,109 Nepalese immigrants with 57.3% being males, and a majority fall within the working-age ranged between 25 to 35 years [34]. A sizeable 33% are dependents, while 20% hold technical and international services visas, 17% are on study visas, 12% possess skilled visas, and 6% have obtained permanent residency (PR) status [35]. Regarding work status, around 30.1% are engaged in the food and beverage industry, 23.7% are employed in other service sectors, 16.8% are involved in wholesale and retail trade, and 13.2% work in the manufacturing sector [36]. The migrants, particularly those with low proficiency in English or Japanese, may face challenges accessing health services during the COVID-19 crisis.

Furthermore, lockdowns and isolation also limit the ability of migrants to gather and speak up about their concerns, and affected their employment status, requiring access to social welfare and support services which may not have been tailored to non-Japanese recipients. Therefore, a thorough, comprehensive assessment is needed to evaluate the diverse problems experienced by migrants during the COVID-19 crisis in Japan. This study aimed to identify barriers associated with access to healthcare due to the COVID-19 crisis with Nepalese migrants as subjects of the study and explore ways to counter these barriers, both in routine and crisis situations.

## Methods

The study employed a mixed methods approach to examine barriers and facilitating factors in healthcare accessibility. By combining qualitative and quantitative methods, the researchers aimed at obtaining a comprehensive understanding of these factors while triangulating the findings. An exploratory sequential design was chosen, allowing for a qualitative exploration of barriers and facilitators among migrants followed by quantitative analysis to assess the generalizability of the themes to the larger population.

Eleven Focus Group Discussions (FGD) were conducted with a total of 89 participants via Zoom. Purposive and snowball sampling techniques were used to recruit participants. Participants included Nepalese aged 18 years and above who had been living in Japan for at least six months and were not on a refugee visa status. It is to note that the FGDs were conducted in August and September 2021, and most participants had either received a single dose of COVID-19 vaccines or not received them due to supply shortages and scheduling difficulties. The FGD questionnaire was conceptually based on the Health Care Access Barrier (HCAB) model of Carrillo et al. [37] Participants were encouraged to express their experiences related to accessing healthcare, what they did if they fell ill, who they approached, how they sought information, any financial issues they had, their opinions of the Japanese healthcare system, opinions on COVID-19, experience of vaccinations, their relationships with schools or employers, coping experience, any factors that they found helpful to overcome any difficulties they had in accessing healthcare, and any recommendations for improving healthcare access to better prepare for the future. All FGDs were conducted by the authors as moderator (SP) and facilitator (AKB). Data was initially coded by generation of numerous category codes without limiting the number of codes [38]. The researchers the used focused coding to eliminate, combine, or subdivide the coding categories identified in the first step. Keywords were identified as indicators of important themes of barriers and facilitators to healthcare access during the COVID-19 crisis. Data saturation was said to occur when the researcher had obtained the full range of ideas and no new information was obtained from having another FGD [39]. Data saturation was obtained within eight FGDs.

After the FGD, cross-sectional correlation research design was conducted to evaluate how easy or difficult it was for people to access healthcare during the COVID-19 outbreak, and what factors helped or refrained them in doing so. Sample size for the study was determined considering an estimated proportion (p) as 50% owing to health accessibility, at 5% margin of error (d). Based on these assumptions, using the sample size formula *n* = z^2^p (1 − p)/d^2^, a minimum sample size of 384 was calculated [40–42].

The survey instrument contained a series of questions based on the themes derived from the FGDs conducted in the first phase of the study. To ensure credibility, the researcher held a meeting with experts, a few representative participants, and Nepali stakeholders residing in Japan to discuss the developed themes. The final questionnaire items were translated in Nepali language both by the researcher and a native professional translating company. 30 Nepalese migrants who did not belong to the focus groups were piloted online to determine the clarity, simplicity, and flow of questions. The final version of the questionnaire was then designed using Google Form to administer it online. The survey form was distributed through social media like Facebook pages and groups, Instagram, Messenger, TikTok, and emails. A total of 1234 responses were received, where 297 were eliminated as invalid or incomplete because respondents stated that they had never used any health services or sought any information during the COVID-19 crisis. Overall, 937 responses who utilized health services were used for the analysis in this study.

The outcome variable was healthcare accessibility, assessed using a binary response question, “Were you able to access health care services during COVID-19 pandemic?” and the independent variables related to the barriers and facilitators were measured in the form of their agreement on a 5-point Likert scale. The scale was rated as 1 = strongly disagree, 2 = disagree, 3 = neutral, 4 = agree, and 5 = strongly agree. However, we recoded the themes into binary category as 0 if people stayed neutral, disagreed, or strongly disagreed to the statement and 1 if they agreed or strongly agreed the statement. The data analysis for the study consisted of descriptive statistics represented by frequencies and percentages, and inferential statistics in the forms of correlations and multivariable logistic regression. Multicollinearity among the independent variables was checked through the variance inflation factor (VIF). SPSS was used for data coding and analysis.

Integration at the methods level was done using the building approach where the item selection for a survey questionnaire was based on previously gathered qualitative data that identifies constructs [43]. Data integration at the interpretation level was done through 'joint display,' which involved displaying the integrated findings in the form of a table [44]. Of the different kinds of joint display, ‘generalizing themes display’ was used. The ‘fit’ of data integration was demonstrated by ‘confirmation’ when the findings confirmed the results, and "discordance," when the findings were contradictory [45, 46]. Ethical approval was obtained from the Independent Research Ethics Review Committee of the University of Kochi in Japan and a web-based informed consent was taken from the participants before the discussions and the survey.

## Results

Sociodemographic characteristics of our participants in both the FGD and survey study are presented in Table 1. The average age of respondents participating in both of our studies were relatively young with mean age of 27.46 ± 4.7 in FGD and 28.35 ± 4.9 in the survey study. More than half of the respondents were female in both the studies with the majority being Hindu. A very small numbers of participants (< 15%) had not completed 12 years of schooling and about 42.7% of our participants in FGD had completed vocational training during their stay in Japan which was even higher among our survey participants (54.9%). Most of the participants in both the studies had working visa status, with more than 40% being full-time employees in Japan, and less than one-fourth of the survey respondents’ income status was affected by the pandemic. About 58% of our survey participants were married. Many of our respondents had lived in Japan for more than five years and the majority had a Japanese and English language ability ranging from fair to good. About 27% of the participants in the FGD had experienced COVID-19 infection and about half were not vaccinated against the infection, but only 20.7% of survey respondents had COVID-19 infection, with more than 90% being fully vaccinated. (Table 1).

**Table 1:** Sociodemographic characteristics of the participants

	FGDs, N (%)	Survey, N (%)
Age		
18–24 years	25 (28.1)	213 (22.7)
25–34 years	55 (61.8)	624 (66.6)
35 above	9 (10.1)	100 (10.7)
Gender		
Male	42 (47.2)	398 (42.5)
Female	47 (52.8)	539 (57.5)
Length of stay in Japan		
6 months to 2 years	15 (16.9)	61 (6.5)
2 years above to 6 years	50 (56.2)	520 (55.5)
more than 6 years	24 (26.9)	356 (38)
Religion		
Hindu	84 (94.4)	814 (86.9)
Others	5 (5.6)	123 (13.1)
Education in Nepal before migration		
12 years of schooling or below	57 (64)	588 (62.7)
Bachelor’s degree and above	32 (36)	349 (37.3)
Education in Japan		
Japanese Language school /Training college	49 (55)	604 (64.5)
University undergraduate and above	15 (16.9)	164 (17.5)
No formal education in Japan	25 (28.1)	169 (18)
Family status in Japan		
Living together with family/ friends	67 (75.2)	626 (66.8)
Living alone	22 (24.8)	311 (33.2)
Visa status		
Student visa	20 (22.5)	293 (31.3)
Working visa/ Skilled Labor visa	37 (41.6)	435 (46.4)
Dependent visa	24 (27)	140 (15)
Business visa	2 (2.2)	22 (2.3)
Permanent resident / Others	6 (6.7)	47 (5)
Employment status		
Full-time employee / Self-employed	39 (43.8)	473 (50.4)
Part-time employee	40 (45)	419 (44.8)
Unemployed / not seeking a job	10 (11.2)	45 (4.8)
Income		
Affected	43 (48.3)	219 (23.4)
Neutral	17 (19.1)	250 (26.7)
Somehow not affected	29 (32.6)	468 (49.9)
English language ability		
Don’t know English at all	2 (2.2)	35 (3.7)
Can understand a basic level of English	54 (60.7)	392 (41.9)
Can do active discussions/express ideas in English	33 (37.1)	510 (54.4)
Japanese language ability		
Don’t know Japanese at all	6 (6.7)	32 (3.4)
Speak and understand: Intermediate level	60 (67.4)	521 (55.6)
Speak and understand: Business/ Native level	23 (25.9)	384 (41)
COVID-19 infection		
Yes	24 (27)	194 (20.7)
No	65 (73)	743 (79.3)
Vaccination status		
Fully Vaccinated (single/two-dose)	44 (49.4)	860 (91.8)
Unvaccinated	45 (50.6)	77 (8.2)
Health insurance		
Yes	89 (100)	887 (94.7)
No	0 (0)	50 (5.3)
Previous experience of a hospital visit		
Yes	80 (89.9)	862 (92)
No	9 (10.1)	75 (8)

*FGDs* Focus Group Discussions, *N* Number of participants

We identified 26 themes of barriers and six themes of facilitating factors based on the Health Care Access Barriers (HCAB) Model from our focus group discussions. Pearson correlation analysis showed a moderate positive correlation between ‘perceived denial of care’ and ‘perception of delay in care’ (*r* = 0.65, *p* < 0.01) and ‘free COVID-19 medical care’ and ‘health insurance’ (*r* = 0.74, *p* < 0.01). (Supplementary file 1) The 'joint display’ is shown in Table 2 which arrays both quantitative and qualitative results. In qualitative analysis, 26 themes of barriers were generated. When quantified to a larger population, the main barrier that respondents agreed with the most was the lack of medical interpreters (68.3%), followed by lack of awareness regarding the health care cost (66.9%) as they were not aware of the health care service utilization cost in Japan. The third was the fear of catching the virus (62.6%). Next was a lack of awareness of health insurance, where respondents agreed that they avoid visiting hospitals thinking that medical health care is expensive in Japan despite paying for health insurance (58.3%).

**Table 2:** Joint display of qualitative themes with quantitative findings of the survey items (*N* = 937)

Category	Themes	Representative sentences	Corresponding survey items	Proportion of agreed	Crude OR (95% CI)	Adjusted OR (95% CI)	Inferences
Financial barriers	Lack of knowledge of health insurance	“I was scared to visit a hospital because I thought the health care cost in Japan is too expensive. When my symptoms got worse, I had a checkup. I was surprised to see that my bill was less expensive than I expected it to be” (FN-1a)	FN1a. I think healthcare costs are expensive in Japan	58.30%	0.54 (0.41—0.70) ***	0.55 (0.42—0.74) ***	Confirmation
		“In Nepal, I had no insurance. I did not know the benefits of having health insurance” (FN-1b)	FN1b. I didn’t/don’t know the benefits of having health insurance	35.40%			
		“I have chronic lung disease. I have been taking medicines from Nepal. During my stay in Japan, I have realized that it is much easier to get treatment if I say I have symptoms, and I have to onegai (request) a lot if I have no symptoms” (FN-1c)	FN1c. It is difficult to get treatment if there are no symptoms in the body	56.10%			
		“Health insurance is a waste of money because I have never used health services” (FN-1d)	FN1d. Insurance is a waste of money	22.80%			
	lack of knowedge on the health care service utilization cost	“There were some services which were covered by health insurance, and some were not covered by health insurance. So, I worried what if my bills were too high” (FN-5a)	FN2. Even with insurance, it is difficult to decide what care is affordable and what is not because the price is unknown	66.90%	1.04 (0.79—1.37)	1.13 (0.84—1.51)	Discordance
		“I did not know how much we needed to pay for healthcare even if I had health insurance. It was difficult to decide which care was affordable and which was not because the price of the care and service provided was not known. I wondered what if medicines were expensive or my CT scanning was expensive, it was scary to me as I had limited budgets” (FN-5b)					
Structural barriers	Perceived denial of care	“I was having difficulty in breathing and my blood oxygen saturation was dropping. I called the public health center many times, but I was being repeatedly denied that I was not sick enough to get hospitalized. Later, I pretended to be unconscious and asked my friend to speak on behalf of me, consequently, I got admitted” (S-1a)	S1. Hospital/ clinic/ public health centers denied providing healthcare services	23.10%	0.86 (0.64—1.18)	0.92 (0.65—1.28)	Discordance
		“My health condition was not good. I called hokenjyo (public health center). They told me to do gaman (patience) and said matte (to wait). Then I called a health clinic nearby. They told me that this was a COVID-19 case and they could not accept me. They told me to call hokenjo (public health center) again” (S-1b)					
		“After vaccination, I had a high-grade fever. I was taking medicines at home, but my fever was not subsiding. After 3 days, I went to the hospital, however, the hospital refused to admit me. I was told that admission was unnecessary despite my unwellness” (S-1c)					
	Perceived delay in care	“It took two days for the health center to provide me medicines for fever” (S-2a)	S2. The healthcare service was late	23%	0.63 (0.46—0.87) **	0.63 (0.45—0.89) **	Confirmation
		“I had to call the public health center so many times. After a week healthcare providers came to my house” (S-2b)					
		“It was difficult to call the health center. The line was too busy. I could not make it. So, I called a Nepali nurse in Japan and took drug stores medicines that she recommended” (S-2c)					
	Limited operating hours of hospitals	“We took our friend with a mental health emergency to hospital, but they told us to come tomorrow as the operating hour was already over for the day. We could not find other hospitals either. We waited until the next morning” (S-3)	S3. Limited opening hours of the hospitals delayed emergency care	35.30%	0.65 (0.50—0.86) **	0.60 (0.45—0.81) **	Confirmation
	Temporary disruption in healthcare services	“Fever medicines were not available at drugstores” (S-4a)	S4. Hospital beds/ Ambulance/were Medicines for COVID-19 were not available	20.10%	0.81 (0.59—1.12)	0.80 (0.56—1.14)	Discordance
		“Hospital beds were not available. I got treatment inside ambulance” (S-4b)					
		“I tried to call ambulances, but they were not available” (S-4c)					
	Perceived complexity in vaccine registrations	“Vaccines were not available. I tried to register my name for the vaccine but each time the person on the phone said sumimasen (sorry), there were no vacancy centers. I got COVID-19 infection later” (S-5a)	S5. It is/was difficult to make a reservation for vaccination	33.30%	0.63 (0.48—0.84) **	0.60 (0.44—0.82) **	Confirmation
		“I tried calling so many times but the call was not accessible” (S-5b)					
		“I had to call every week to ensure that I would get registered. It was then (difficult).” (S-5c)					
	Lack of medical interpreters	“I knew some hospitals that provided the English interpreters, but I did not know the one with Nepali interpreters. I could not explain my medical condition properly neither in English language nor in the Japanese language. I usually asked my friend to help me visit the hospital. Otherwise, I did not go alone.” (S-6a)	S6. Medical interpreters were not available	68.30%	0.73 (0.55—0.97) *	0.70 (0.52—0.94) *	Confirmation
		“I could understand the Japanese language, but I could not understand the terminology. There were no medical interpreters.” (S-6b)					
	Lack of the hotline services	“My city provided COVID hotline services, however, it was not accessible when needed. Each time I called the center, the call was busy.” (S-7a)	S7. COVID-19 Hotlines were not available when needed	27.40%	0.68 (0.51—0.92) *	0.67 (0.49—0.92) *	Confirmation
		“Most of the information I got was from the social media. I wish there was a number I could dial and ask, but I didn’t know where to call.” (S-7b)					
Cognitive barriers	Unawareness of available services	“In Japan, I did not know where to go if I had some diseases” (COG-1a)	COG1. I didn’t/don't know where to go if I have health problems	35.10%	0.44 (0.33—0.59) ***	0.49 (0.36—0.66) ***	Confirmation
		“Having lived already for three years in Japan, I finally knew about the mental health service hotline available in the Nepali language” (COG-1b)					
	Difficulty in trusting healthcare providers	“I was pregnant. My doctor told me that it was safe to take the vaccines, but I had already heard that some doctors refused to administer the vaccines to pregnant women. I did not know whom to trust. I did not want anything to happen to my baby” (COG-2a)	COG2. I don’t know whom to trust because different doctors have different opinions	32.20%	0.66 (0.50—0.86) **	0.64 (0.47—0.86) **	Confirmation
		“Some scientists and doctors said that the COVID-19 was not real, while other doctors said it was real. Why did doctors have different opinions? Both were doctors. Who should I believe?” (COG-2b)					
	Information overload	“There was much information about the preventive measures of COVID-19. We could not follow each and everything, like social distancing. I felt overloaded and I did not want to hear the same thing again and again” (COG-3a)	COG3. Too much information from different sources made confusion	40.60%	0.70 (0.54—0.91) **	0.70 (0.53—0.93) *	Confirmation
		“All over the world the information-related media were talking about the COVID-19 and it was too much for me to hear the same thing every day. Some used to say the beta virus was dangerous and now they say this delta is dangerous. I think there is no end to COVID-19 because it is just like an influenza virus. There is no need to worry” (COG-3b)					
		“I had Covid-19 infection last month. Some said that we could take the vaccine after 14 days; while others said we could take the vaccine after 30 days; and few others said that there was no need to take vaccines for the next two to three months. It was so confusing. Please clarify” (COG-3c)					
	Limited awareness of COVID-19 and vaccination	“I didn't know sign in symptoms of COVID-19” (COG-4a)	COG4a. I didn’t/don’t sign and symptoms of COVID-19	21.10%	0.56 (0.43—0.73) ***	0.58 (0.44—0.78) ***	Confirmation
		“I didn’t know how vaccines work and whether I was liable to be t vaccinated or not.” (COG-4b)	COG4b. I didn’t know how vaccines work and whether I am liable to get vaccinated or not	30.10%			
		COVID-19 is a game to reduce the world's population.” (COG-4c)	COG4c. COVID-19 is a game to reduce the world's population	27.90%			
Cultural barriers	Language barriers	“I did not know the language” (CUL-1a)	CUL1. I had language problems	37.20%	0.48 (0.36—0.63) ***	0.51 (0.38—0.70) ***	Confirmation
		“What to say when I called to the clinic or hospital” (Cul-1b)					
		“I wanted to go to hospital, but I was not confident enough about my language ability to speak up with doctor” (CUL-1c)					
	Communication barriers	“Doctors did not talk clearly about the diseases. They used to say kamosirenai (maybe), machimasyo (let’s wait). Hakkiri iutte hosii na (I wished doctors would tell me clearly)” (CUL-2)	CUL2. Doctors don't tell us clearly about the disease	31.30%	0.56 (0.42—0.75) ***	0.55 (0.40—0.75) ***	Confirmation
	Self-healing practices	“I waited for self-healing. I had ginger, turmeric, honey, and basil leaves which relieved me a bit for my throat pain. Later my fever was not decreasing so I asked a Nepali senpai (senior) and called the public health Center” (CUL -3a)	CUL3. I waited for self-healing	37.50%	0.70 (0.53—0.92) **	0.77 (0.57—1.03)	Discordance
		“I wanted to heal myself at first; and if the disease got severe then I would like to visit the hospital” (CUL-3b)					
	Cultural food preferences	“I didn't eat the food at the hospital because I preferred Nepali food. It was difficult to stay at the hospital without good food” (CUL-4a)	CUL4. Hospital/Hokenjo food was not tasty / not preferable	30.60%	0.80 (0.59—1.03)	0.79 (0.58—1.08)	Discordance
		“The food from hokenjyo (public health center) was not tasty, so I asked some of my friends to bring food for me (CUL-4b)					
		“I used to get sick more in a hospital because I could not eat food at all” (CUL-4c)					
		“Hospital/Hokenjo food was not preferable” (CUL-4d)					
	Inefficacy of low- dose drug	“I usually bring my medicines from Nepal which work because medicines in Japan don’t work like medicines in Nepal” (Cul-5)	CUL5. Medicines in Japan don't work like medicines in Nepal	22.40%	0.65 (0.47—0.90) **	0.67 (0.48—0.94) *	Confirmation
	Unfamiliarity with Japanese medical system	“The Japanese medical system was difficult to understand” (Cul-6a)	CUL6. The Japanese medical system is difficult to understand	34.30%	0.51 (0.38—0.67) ***	0.52 (0.38—0.70) ***	Confirmation
		“In Nepal, we have a culture of natural healing and herbal medicines. Neither there are many health facilities in Nepal nor there is health insurance system. When I came to Japan for the first time, I came to know that there were different departments of health services. We needed to book for services or care provided here which was not a usual practice in our country. There are many papers works to do. Many hospitals are closed on holidays. The system is different” (Cul-6b)					
Psycho-logical barriers	Fear of catching virus	“I feared catching the virus, so I got scared to go to hospitals. I even got fear of catching the virus from clothes of people inside trains” (P-1)	P1. I had fear of catching the virus	62.60%	1.01 (0.77—1.31)	1.15 (0.86—1.54)	Discordance
	Fear of losing job	“If I had to go to the hospital then I could not have done my part-time work. I had to leave the job, but I needed money. The staff was also very few. I did not want to leave or lose my job.” (P-2)	P2. I had fear of losing my job	49.30%	0.64 (0.49—0.83) **	0.74 (0.56—0.99) *	Confirmation
	Fear of discrimination	“I had been hearing news about discrimination at many places and even in other countries. I had heard of threatening and deportation, so I had also fear of discrimination” (P-3a)	P3. I had fear of discrimination	45.50%	0.50 (0.39—0.66) ***	0.57 (0.43—0.76) ***	Confirmation
		“I could realize the differences in the information provided for Japanese and for migrants in my company. I felt as if my manager did not like to speak to me. I don't know if this was a culture or whatsoever, but I felt discriminated. So, I do not want to share about my health issues” (P-3b)					
	Privacy issues	“I did not want to share my information at that time. The time has changed but at that time, I wanted my privacy. People would believe that I got Covid-19 due to my negligence.” (P-4)	P4. I had my privacy concerns	27.80%	0.74 (0.55—0.99) *	0.83 (0.60—1.13)	Discordance
	Loneliness	“I stayed for 15 days without my baby and my husband, and I was lonely.” (P-5a)	P5. I was lonely or afraid of being lonely at the hospital	31.30%	0.69 (0.52—0.92) *	0.74 (0.55—1.01)	Discordance
		“I did not want to go to the hospital because my family could not stay with me together” (P-5b)					
	Depression due to loss in income	““My income has been reduced. My company gives me less money. I needed money for my gakko (school). I was depressed” (P-6)	P6. I was/am depressed due to a loss in income	41.20%	0.71 (0.55—0.93) *	0.89 (0.67—1.20)	Discordance
Legal barriers	Problems with legal documentation	“I was a migrant to this city, and I had my documents in another prefecture. My company refused to give me the shot. It was not even possible at that time to go back to my prefecture to get the documents” (L-1)	L1. I had problems due to legal documentation	24.70%	0.60 (0.44—0.82) **	0.61 (0.44—0.86) **	Confirmation
Facilitators	Free COVID-19 medical care	“I was infected with COVID 19 and was admitted to hospital. At first, I was worried about medical expenses, but I was not required to pay the bills except for my expenses like drinks and snacks. Free medical care became a blessing for me in Japan” (FA-1a)	FA1. COVID-19 free medical service from the government was helpful	73.90%	1.33 (0.99—1.79)	1.22 (0.88—1.68)	Discordance
		“My husband lost his job when he got admitted to hospital. We were not in a position to manage hospital expenditures. Although we had to pay for transportation to the hospital, we did not pay for hospital expenditure. At least, free healthcare service became helpful” (FA-1b)					
		“Some medical workers came at my home, they checked-up and gave medicines for free” (FA-1c)					
	Health insurance	“Actually, my friend told me about the health insurance policy. He made me realize that I had also paid for health insurance, so I need to pay 30% if I go for a checkup. At that time, I had problems with the tingling sensation in my hands. I had to do different nerves tests. I thought that the bills of those procedures would be too expensive, but it was 3 or 4 thousand in total, which I could afford. It was less expensive than my expectation” (FA-2a)	FA2. Having national health insurance was helpful	70.70%	1.17 (0.88—1.57)	1.20 (0.88—1.63)	Discordance
		“I have insurance from the company. Health insurance is helpful for us, so I encourage everyone to pay insurance premium monthly” (FA-2b)					
	Medical interpreters	“I know a clinic where there is a Nepali sister as an interpreter. I used to go that clinic. I also got my vaccination in that clinic with help of her” (FA-3a)	FA3. Medical interpreters were available	27.90%	0.97 (0.73—1.30)	1.05 (0.77—1.43)	Discordance
		“Mother of my friend was recently diagnosed with lung cancer. The doctor had requested to find an interpreter. I helped her family in searching clinics that had Nepali medical interpreters, but when we could not find, the hospital arranged it by themselves” (FA-3b)					
	Mutual aid	“When I became COVID-19 positive, I had to stay in quarantine. I live alone but my friends helped me by bringing foods and medicines for me” (FA-4a)	FA4. Friends/family/school/company/ helped me get food/medicines/money	62.20%	1.29 (0.99—1.67)	1.32 (1.00—1.74)	Discordance
		“I was mentally depressed because I could not pay school fees due to loss in income. The aid given was not sufficient. Gakko (school) also gave some flexibilities by providing scholarship, and my friends helped me by lending some money for my livelihood” (FA-4b)					
		“When I was in quarantine, my company helped me giving COVID test kits, even food and other materials” (FA-4c)					
	Telehealth services	“I saw information on Facebook about some Nepali doctors providing free medical consultations for those infected with COVID-19 and taking care of their health at home. I called the number, and it was free. The doctor consulted me on zoom. I was advised to take medicines from drugstores he prescribed” (FA-5a)	FA5. Telehealth services with doctors was helpful	30.30%	1.05 (0.79—1.39)	1.00 (0.73—1.36)	Discordance
		“When my whole family in Nepal was in isolation due to COVID-19 infection, they could access Nepali doctors and nurses online. Later when I got COVID-19 here in Japan, I consulted with one of the same doctors online. It was helpful for me in Japan too” (FA-5b)					
		“I called a public health doctor in the US using Viber app” (FA-5c)					
	Health information from Nepali nurses/doctors and health volunteers	“There was a zoom seminar about COVID-19 information from a group of medical personnel living in Japan, broadcasted live on many Nepali online news channels of Japan. That seminar was helpful to understand preventive measures, self-management at home for mild symptoms, and about vaccination” (FA-6a)	FA6. Health information from Nepali nurses/doctors/health volunteers was helpful	61.20%	1.27 (0.97—1.66)	1.36 (1.01—1.82) *	Confirmation
		“People were saying that vaccine is harmful, it is just being tested in human. It was a confusing state for me who do not understand what was going on. However, I trusted a Nepali nurse who was living in Japan and was sharing COVID-19 related informative videos daily on her social media, that was helpful to me” (FA-6b)					
		“Nepali Association volunteers helped me get my vaccination shot” (FA-6c)					
		It was difficult to call the health center. The line was too busy. I could not make it. So, I called a Nepali nurse in Japan and took drug stores medicines that she recommended” (FA-6d)					

*OR* Odds Ratio (obtained from logistic regression analysis), *CI* Confidence Interval, Adjusted *OR* Odds ratio of disagreement compared to the agreement upon respective barriers or facilitators obtained after adjusting the sociodemographic characteristics of the survey participants, *p-value < 0.05, **p-value < 0.01, ***p-value < 0.001

Nepalese respondents agreed that there were many facilitators to help them access health care services in Japan. In the qualitative analysis, six themes of facilitators were identified. When quantified to the larger population, the majority agreed that the main facilitator was free COVID-19 medical care from the Japanese Government (73.9%), followed by having health insurance coverage system (70.7%). The next facilitator was mutual aid (62.2%) followed by the web-based health information provided by Nepali doctors, nurses, and health volunteers (61.2%). The least agreed facilitator was the availability of medical interpreters when needed (27.9%). Some of these results were also confirmed by our multivariable logistic regression analysis which identified the odds ratio of agreement upon the barrier or facilitator comparing those who have poor or better health care accessibility after adjusting for all covariates under analysis. We found that compared to those who have poor accessibility those who have good accessibility were 45% (OR = 0.55, 95% CI = (0.42 – 0.74), *p*-value < 0.001) less likely to agree that theme 1 (“Financial barrier”) was a barrier to health care accessibility after adjusting for all confounders. Similarly, those with good accessibility were less likely to agree that perceived delay in care (OR = 0.63, 95% CI = (0.45 – 0.89), *p*-value < 0.01), limited operating hours of the hospital (OR = 0.60, 95% CI = (0.45 – 0.81), *p*-value < 0.01), perceived complexity in vaccine registration (OR = 0.60, 95% CI = (0.44 – 0.82), *p*-value < 0.01), lack of medical interpreters (OR = 0.70, 95% CI = (0.52 – 0.94), *p*-value < 0.05), lack of hotline services (OR = 0.67, 95% CI = (0.49 – 0.92), *p*-value < 0.05), all cognitive barriers, language barriers (OR = 0.51, 95% CI = (0.38 – 0.70), *p*-value < 0.001), communication barriers (OR = 0.55, 95% CI = (0.40 – 0.75), *p*-value < 0.001), inefficiency of low dose drug (OR = 0.67, 95% CI = (0.48 – 0.94), *p*-value < 0.05), unfamiliarity with Japanese medical system (OR = 0.52, 95% CI = (0.38 – 0.70), *p*-value < 0.001), fear of losing job (OR = 0.74, 95% CI = (0.56 – 0.99), *p*-value < 0.05), fear of discrimination (OR = 0.57, 95% CI = (0.43 – 0.76), *p*-value < 0.001) and legal barriers (OR = 0.61, 95% CI = (0.44 – 0.86), *p*-value < 0.01) were the barriers to health care accessibility. Additionally, participants who have good accessibility of health care had higher likelihood of agreeing on every theme of the facilitators compared to their counterparts however, only receiving health information from Nepali doctors/ nurses and health care volunteers was statistically significant after controlling for all confounders under analysis (OR = 1.36, 95% CI = (1.01 – 1.82), *p*-value < 0.05). (Table 2).

## Discussion

This mixed-method study explored the barriers to and facilitators of healthcare accessibility among Nepalese migrants during the COVID-19 crisis in Japan. This study identified a lack of awareness about the health care system and health insurance coverage in Japan as one of the significant barriers to healthcare access. In addition to literacy, this barrier may also be due to a lack of experience in using health insurance in their home country, Nepal, which still lacks a fully developed national health insurance system [47, 48]. The next barrier identified was the lack of awareness of health care service utilization cost. If people are not aware of the costs of healthcare services before they receive them, they may be more hesitant to pursue treatment due to worries about the potential bills they may have to pay. Studies have shown that price transparency can effectively help to reduce healthcare costs, [49] so more research is needed to explore clinical strategies that can increase awareness on both medical cost and quality among consumers.

It is also important to note that during the COVID-19 pandemic, the healthcare system was constantly changing [50]. Public health centers were overwhelmed by the number of cases and thousands of patients were not able to get treatment in time due to the delayed emergency care or were denied treatment due to lack of beds [51]. The vaccines were not available in time and when it did many people had to wait for months to get the vaccination which might have been the reason that almost half of our population were not vaccinated during the study period. Similarly, the participants viewed limited hospital hours and the lack of a COVID-19 hotline services significantly impacted their perception of difficulty in healthcare access. During a pandemic, sources providing accurate information can help to reduce panic and confusion among people. There is also an existing language barrier due to the lack of Japanese proficiency among migrant populations, making it difficult for them to access health services. The scarcity of medical interpreters and translators exacerbates the problem. A survey done by the Foreign Medical Measures Committee in 2018 reported that, of the 5,611 hospitals that responded, 94.9% did not have medical interpreters [52]. Additionally, the complex Japanese writing system presents further difficulties. Even those who are proficient in Japanese or English may struggle with medical terminologies and expressing their symptoms accurately. ‌Despite government efforts to address the issue, it remains an ongoing challenge due to the continual movement of people from diverse linguistic backgrounds. In addition to medical interpreters and translation apps, incorporating the existing free Nepalese telehealth services could help to overcome this issue. One of the least agreed upon and not significant, yet still noteworthy, barriers were the complaints about medications in Japan. According to Kinuko, S., a Japanese nurse who conducted a study on pain management, many Brazilian women living in Japan had a similar experience in that the anesthesia and sedatives administered after childbirth did not work as expected [53]. This suggests that healthcare policymakers and practitioners should pay attention to the need for personalized medical therapy rather than simply providing an "equivalent" medicine, as genetic variability, dietary patterns, exercise habits, environmental factors like climate, socio-cultural and psychosocial influences, smoking and alcohol consumption can have a major impact on drug metabolism and absorption [54]. Moreover, education about Japanese prescribing policies is important. While doctors typically prescribe lower doses of pain medication initially and adjust dosage based on response and tolerance, this approach can vary. The cultural concept of “Gaman” (Tolerance) may also lead to under-prescription of pain relief medication unless deemed necessary [55].

Fear of infection, job loss and discrimination were identified as psychological barriers to healthcare access. The impacts of the COVID-19 pandemic have been extreme, damaging public mental health [56]. Further in-depth investigation is needed to understand these delicate problems and the types of psychological interventions available in the Japanese context. Furthermore, legal barriers which include administrative obstacles such as paperwork and registration procedures can be overwhelming. Although welfare support was widely accessible in Japan in 2020 and 2021, the support system relied on paper documents, lacked transparency, and was only provided in the Japanese language. This could have led to numerous difficulties for foreigners who had trouble understanding and navigating the system, ultimately preventing them from obtaining the necessary support [57].

The most identified facilitator of healthcare access was free COVID-19 medical care. The country's national health insurance system and other forms of insurance have helped migrants gain access to a variety of healthcare services. Some focus group participants mentioned that they received free consultations with Nepali doctors and nurses in the form of Zoom video calls or telephone. Telehealth services provided by the Non-Resident Nepali Association (NRNA) have also helped meet the health needs of the eight million migrant Nepali workers around the world [58]. Similarly, during times of disasters or isolation, the sharing of resources such as food, medicine, and financial aid has been shown to help in minimizing the effects of disasters [59]. Lastly, the study identified that obtaining health information from Nepali doctors, nurses, and healthcare volunteers was a significant facilitating factor. This finding aligns with the broader recognition of migrant health professionals as valuable assets in addressing the increased healthcare demand during the COVID-19 pandemic in many countries [60].

The rising population of foreigners in Japan, coupled with the anticipated impact of mega-disasters, necessitates the adoption of proactive policies to safeguard the health and safety of migrants, particularly during times of crises or emergencies. It is recommended that the Japanese Ministry of Health and concerned authorities adopt flexible policies to empower and mobilize native migrant healthcare professionals, enabling their effective response during mega-disasters. Collaboration between the Nepal embassy, Nepali associations, local governments, and the central government is crucial in establishing a crisis center that provides timely information and assistance to Nepali citizens in Japan during emergencies. Furthermore, comprehensive pre-migration information on the host country's health insurance system and guidance on choosing appropriate healthcare providers should be provided. Consideration should also be given to making health insurance documentation mandatory during the visa issuance process. Furthermore, offering accessible hotline services in the Nepali language, exploring alternative policies to reduce excessive paperwork during crises, and strengthening primary healthcare services are also crucial steps to ensure inclusive and efficient healthcare provision for migrants in Japan.

### Limitations

Though this was the first mixed method study to identify the barriers to and facilitators of health care accessibility among Nepalese immigrants during the COVID-19 pandemic, it has several limitations. One of the researchers was an influencer actively sharing educational content for Nepalese residing in Japan on social media hence, the participation rate might have been influenced by this. However, the questionnaire developed was anonymous hence, the researcher was not aware of the respondents’ details. Meanwhile, participants were recruited from social networking sites, it could be possible that those who responded to the survey were already willing to learn new ideas which might have affected the findings of the study. However, we received responses from various socioeconomic groups of people and were able to get responses nearly three times than our expectation. Hence, the results could be generalized to the Nepali immigrants residing in Japan. Also, this study was a cross-sectional study, and causal conclusions cannot be drawn. It was possible that the aid or facilitating factors identified in this study were temporary and may have changed over time as the pandemic changed. For this purpose, a longitudinal study design needs to be employed by future studies to determine the cause and effect in the study area over time.

## Conclusion

During a global-scale pandemic like COVID-19, migrating populations are likely to face increased burden. Our study highlighted on the barriers and facilitating factors affecting healthcare accessibility for Nepalese migrants in Japan during the crisis. Through focus group discussions and survey analysis, we identified a range of barriers including limited knowledge of health insurance, language barriers, unawareness of available services, and so on. Without mixed-methods data integration, the identification of important but least agreed barriers such as inefficacy of low-dose drugs and telehealth services would have been shadowed. The impact of low-dose drugs and the global availability of free Nepali telehealth services during the COVID-19 crisis cannot be ignored, and both findings deserve further in-depth investigations. The findings also provide crucial insights into the structural challenges faced by migrant communities, emphasizing the need for policy interventions. To enhance future disaster preparedness, we recommend the establishment of a crisis information hub by the Nepal Embassy or concerned authorities, active deployment of Nepalese health workers and volunteers, availability of hotlines, and incorporation of telehealth services. By addressing these barriers and capitalizing on facilitating factors, we can create a more inclusive healthcare system that ensures the well-being of Nepalese migrants during the crisis situation and beyond. Further research is also needed to explore the experiences of other migrant groups.

## Supplementary Information

**Additional file 1.** **Table 1.** Correlation between different barriers and facilitators of health care accessibility.

## Data Availability

The questionnaire and dataset used and/or analyzed during the current study are available from the corresponding author only on reasonable request as they are mostly in the native language of Nepal.

## Abbreviations

EHI: Employees' Health Insurance
NHI: National Health Insurance
FGD: Focus Group Discussion
VIF: Variance Inflation Factor
NRNA: Non-Resident Nepali Association
CI: Confidence Interval
OR: Odds Ratio
SNS: Social Networking Site
