# Supplementary material for: Barriers and facilitating factors to healthcare accessibility among Nepalese migrants during COVID-19 crisis in Japan: an exploratory sequential mixed methods study
Source: BMC Public Health. 2023 Jun 24;23:1226. doi: 10.1186/s12889-023-16107-7 (PMC10290307; doi:10.1186/s12889-023-16107-7)
Supplement: Supplementary file 1 — Additional file 1. Table 1. Correlation between different barriers and facilitators of health care accessibility. [file 12889_2023_16107_MOESM1_ESM.docx]

Table 1. Correlation between different barriers and facilitators of health care accessibility

| Variables | 1 | 2 | 3 | 4 | 5 | 6 | 7 | 8 | 9 | 10 | 11 | 12 | 13 | 14 | 15 | 16 | 17 | 18 | 19 | 20 |
| --- | --- | --- | --- | --- | --- | --- | --- | --- | --- | --- | --- | --- | --- | --- | --- | --- | --- | --- | --- | --- |
| 1. S1 | 1 |  |  |  |  |  |  |  |  |  |  |  |  |  |  |  |  |  |  |  |
| 2. S2 | .**65**** | 1 |  |  |  |  |  |  |  |  |  |  |  |  |  |  |  |  |  |  |
| 3. S3 |  | .56** | 1 |  |  |  |  |  |  |  |  |  |  |  |  |  |  |  |  |  |
| 4. S4 | .61** | .62** | .58** | 1 |  |  |  |  |  |  |  |  |  |  |  |  |  |  |  |  |
| 5. COG1 |  |  |  |  | 1 |  |  |  |  |  |  |  |  |  |  |  |  |  |  |  |
| 6. COG2 |  |  |  |  | .53** | 1 |  |  |  |  |  |  |  |  |  |  |  |  |  |  |
| 7. COG3 |  |  |  |  |  | .67** | 1 |  |  |  |  |  |  |  |  |  |  |  |  |  |
| 8.COG4 |  |  |  |  | .52** |  |  | 1 |  |  |  |  |  |  |  |  |  |  |  |  |
| 9.CUL1 |  |  |  |  |  |  |  |  | 1 |  |  |  |  |  |  |  |  |  |  |  |
| 10.CUL5 |  |  |  |  |  |  |  | .51** | .50** | 1 |  |  |  |  |  |  |  |  |  |  |
| 11.CUL6 |  |  |  |  |  |  |  |  | .56** | .56** | 1 |  |  |  |  |  |  |  |  |  |
| 12. P1 |  |  |  |  |  |  |  |  |  |  |  |  |  |  |  |  |  |  |  |  |
| 13. P2 |  |  |  |  |  |  |  |  |  |  |  | .51** | 1 |  |  |  |  |  |  |  |
| 14. P3 |  |  |  |  |  |  |  |  |  |  |  |  | .54** | 1 |  |  |  |  |  |  |
| 15. P4 |  |  |  |  |  |  |  |  |  |  |  |  |  | .59** | 1 |  |  |  |  |  |
| 16. P5 |  |  |  |  |  |  |  |  |  |  |  |  |  | .55** | .57** | 1 |  |  |  |  |
| 17. P6 |  |  |  |  |  |  |  |  |  |  |  |  |  | .50** |  |  | 1 |  |  |  |
| 18. L1 |  |  |  |  |  |  |  | .51** |  |  |  |  |  |  |  | .50** |  | 1 |  |  |
| 19. FA1 |  |  |  |  |  |  |  |  |  |  |  |  |  |  |  |  |  |  | 1 |  |
| 20. FA2 |  |  |  |  |  |  |  |  |  |  |  |  |  |  |  |  |  |  | .74** | 1 |
| 21.Access |  |  |  |  |  |  |  |  |  |  |  |  |  |  |  |  |  |  |  |  |

* p<0.05, ** p<0.01, S1=Perceived denial of care, S2= Perceived delay in care, S3= limited operating hours of hospitals, S4= Temporary disruptions in healthcare services,  COG1= Unawareness of available services, COG2= Difficulty in trusting healthcare providers, COG4= Limited awareness COVID-19 and vaccination CUL1= Communication barriers, CUL5= Inefficacy of low-dose drugs, CUL6= Unfamiliarity with Japanese medical system, P1= Fear of catching the virus, P2= Fear of losing a job, P3= Fear of discrimination, P4= Privacy issues, P5= Loneliness, P6= Depression due to loss in income, L1= Problems with the legal documentations, FA1= Free COVID-19 medical care, FA2= Health insurance
